# Frequent loss of expression or aberrant alternative splicing of P2XM, a p53-inducible gene, in soft-tissue tumours

**DOI:** 10.1038/sj.bjc.6690484

**Published:** 1999-06

**Authors:** G Nawa, Y Miyoshi, H Yoshikawa, T Ochi, Y Nakamura

**Affiliations:** Division of Clinical Genetics, Department of Medical Genetics, and Department of Orthopedic Surgery, Biomedical Research Center, Osaka University Medical School, Suita-shi, Osaka, 565-0871, Japan; Department of Orthopedic Surgery, Osaka Medical Center for Cancer and Cardiovascular Diseases, 1-3-3 Nakamichi, Osaka-shi, Osaka, 537-0025, Japan; Laboratory of Molecular Medicine, Human Genome Center, The Institute of Medical Science, The University of Tokyo, Minato-ku, Tokyo, 108-8639, Japan

**Keywords:** P2XM, alternative splice, soft tissue sarcoma, p53, mdm2

## Abstract

We investigated expression of a human p53-inducible gene, P2XM, a member of the P2X-receptor family of ATP-gated ion channels, in 56 human primary soft-tissue tumours including 47 sarcomas and nine benign tumors. Among the 47 sarcomas examined by reverse transcription polymerase chain reaction, 12 had lost expression of this gene and 22 revealed altered splicing patterns; among the nine benign tumours, four showed no expression of P2XM and three revealed aberrant splicing patterns involving transmembrane domains M1 and/or M2. As the aberrant transcripts lacked either or both of those domains, the protein products probably lacked normal function. We also looked for p53 mutations and mdm2 overexpression in the same panel of tumours and found them in 13 tumours, all but three of which had shown altered expression of P2XM. However, 31 (72%) of the 43 tumours that carried wild-type p53 without mdm2 overexpression had revealed aberrant P2XM expression. Our results suggest that disorder of P2XM expression may play a crucial role in the genesis of benign and malignant tumours in soft tissues, and that one or more genetic factors other than p53 or mdm2 contribute significantly to aberrant P2XM expression. © 1999 Cancer Research Campaign


*British Journal of Cancer* (1999) **80**(8), 1185–1189
© 1999 Cancer Research Campaign
Article no. bjoc.1999.0484

### Frequent loss of expression or aberrant alternative splicing of P2XM, a p53-inducible gene, in soft-tissue tumours

**G Nawa[1,2], Y Miyoshi[1], H Yoshikawa[2,3], T Ochi[2] and Y Nakamura[1,4]**

[1]Division of Clinical Genetics, Department of Medical Genetics and [2]Department of Orthopedic Surgery, Biomedical Research Center, Osaka University Medical School, 2-2 Yamadaoka, Suita-shi, Osaka 565-0871, Japan; [3]Department of Orthopedic Surgery, Osaka Medical Center for Cancer and Cardiovascular Diseases, 1-3-3 Nakamichi, Higashinari-ku, Osaka-shi, Osaka 537-0025, Japan; [4]Laboratory of Molecular Medicine, Human Genome Center, The Institute of Medical Science, The University of Tokyo, 4-6-1 Shirokanedai, Minato-ku, Tokyo 108-8639, Japan

**Summary** We investigated expression of a human p53-inducible gene, P2XM, a member of the P2X-receptor family of ATP-gated ion channels, in 56 human primary soft-tissue tumours including 47 sarcomas and nine benign tumors. Among the 47 sarcomas examined by reverse transcription polymerase chain reaction, 12 had lost expression of this gene and 22 revealed altered splicing patterns; among the nine benign tumours, four showed no expression of P2XM and three revealed aberrant splicing patterns involving transmembrane domains M1 and/or M2. As the aberrant transcripts lacked either or both of those domains, the protein products probably lacked normal function. We also looked for p53 mutations and mdm2 overexpression in the same panel of tumours and found them in 13 tumours, all but three of which had shown altered expression of P2XM. However, 31 (72%) of the 43 tumours that carried wild-type p53 without mdm2 overexpression had revealed aberrant P2XM expression. Our results suggest that disorder of P2XM expression may play a crucial role in the genesis of benign and malignant tumours in soft tissues, and that one or more genetic factors other than p53 or mdm2 contribute significantly to aberrant P2XM expression.

**Keywords:** P2XM; alternative splice; soft tissue sarcoma; p53; mdm2

Soft-tissue sarcomas (STSs) are classified into various histopathological types such as malignant fibrous histiocytoma (MFH), liposarcoma, fibrosarcoma, rhabdomyosarcoma, leiomyosarcoma and malignant schwannoma (Enzinger et al, 1969; Enjoji et al, 1984). However, possible genetic differences among these types, for example what molecular alterations occur commonly or correspond particularly to each tumour type, have not been established.

Mutations of the p53 gene have been detected in STSs (Masuda et al, 1987; Toguchida et al, 1992) as well as in solid tumours from colon, breast and lung. The reported frequencies of p53 mutations in STSs vary: 10–36% in MFH, 11–23% in liposarcoma, 14–38% in leiomyosarcoma (Stratton et al, 1990; Toguchida et al, 1992; Andreassen et al, 1993; Leach et al, 1993; Castresana et al, 1994, 1995; Latres et al, 1994; Taubert et al, 1995, 1996; Würl et al, 1996; Schneider-Stock et al, 1997) and 33% in malignant schwannoma (Schneider-Stock et al, 1997). Stratton et al (1990), Felix et al (1992) and Würl et al (1996) reported that one-third of the rhabdomyosarcomas they examined had p53 mutations, but only a few mutations of that gene have been found in fibrosarcomas (Latres et al, 1994; Taubert et al, 1996). Mutation of p53 is rare in synovial sarcomas (Andreassen et al, 1993; Taubert et al, 1996). Hence the role of p53 mutations in STSs is still unclear. Furthermore, in some tumours carrying wild-type p53, functional inactivation of p53 can also occur through with overexpressed mdm2 which inhibit p53 function by concealing its transcriptional activation domain or destabilizing endogenous p53 protein (Haupt et al, 1997; Kubbutat et al, 1997). It has been reported that the mdm2 gene amplification was observed in more than 30% of human sarcomas (Oliner et al, 1992; Leach et al, 1993; Cordon-Cardo et al, 1994).

The wide variety of biological functions of p53 result from its transcriptional activation of downstream genes such as p21/WAF1, GADD45, GML and BAX (Kastan et al, 1992; Vogelstein and Kinzler, 1992; Wu et al, 1993; Okamoto and Beach, 1994; Buckbinder et al, 1995; Miyashita and Reed 1995; Furuhata et al, 1996; Morris et al, 1996). When p53 functions are suppressed by p53 mutation or overexpression of mdm2, expression of these and unidentified downstream genes, including genes that encode tissue-specific factors as well as common components, are likely to be down- or up-regulated, and their dysfunctional products could be expected to contribute to tumour formation and/or progression. Although a number of p53-target genes have already been isolated, most of them have not been examined intensively for alterations in human primary cancers.

Recently we isolated a human p53-inducible gene, P2XM, encoding a member of the P2X-receptor family of adenosine 5′ triphosphate (ATP) gated ion channels (Urano et al, 1997). Its amino acid sequence contains two predicted transmembrane domains (M1 and M2) and a segment resembling the H5 region of voltage-gated K+ channels that are expressed predominantly in skeletal muscle. In a previous report we described the complete loss of expression of P2XM, or an altered expression pattern in

*Received 8 September 1998*
*Revised 12 January 1999*
*Accepted 27 January 1999*

*Correspondence to:* Y Nakamura, Laboratory of Molecular Medicine, Human Genome Center, The Institute of Medical Science, The University of Tokyo, 4-6-1 Shirokanedai, Minato-ku, Tokyo 108-8639, Japan which alternative splices had removed the transmembrane domains, in cell lines derived from rhabdomyosarcomas or osteosarcomas (Urano et al, 1997). Those findings suggested that loss of normal function of the p53-P2XM signal-transduction pathway might be associated with tumorigenesis in bone or soft tissue.

To address this question, we examined expression of P2XM, as well as the mutation status of p53 and overexpression of mdm2, in 56 primary malignant and benign soft-tissue tumours.

### MATERIALS AND METHODS

### Patients and tissues

We obtained materials from 56 patients (29 males and 27 females) who underwent definitive surgical treatment for soft-tissue tumours at Osaka University Medical School Hospital or at the Osaka Medical Center for Cancer and Cardiovascular Diseases, Osaka, Japan. Of the 47 tumours that were malignant, 15 were diagnosed histopathologically as malignant fibrous histiocytomas (MFHs), nine as liposarcomas, seven as synovial sarcomas, six as malignant schwannomas, three as leiomyosarcomas, two as rhabdomyosarcomas, two as alveolar soft-part sarcomas and three as 'other' or unclassified sarcomas. The ages of the patients at first diagnosis ranged from 6 to 81 years (mean 47.7 years). We also selected nine benign soft-tissue tumours for study, including three lipomas, two desmoid tumours, two schwannomas, one hemangioma and one fibroma.

### RT-PCR of P2XM and mdm2 transcripts

Samples of total RNA extracted from tumour tissues were reversely transcribed to single-stranded cDNAs using oligo(dT)15 primer and superscript II (GibcoBRL, Life Technologies, Rockville, MD, USA). Semiquantitative Polymerase chain reaction (PCR) experiments were performed with cDNA samples whose concentrations were equalized with 20 cycles of PCR amplification of gluceraldehyde 3-phosphate dehydrogenase (GAPDH). The reactions were performed using Ex Taq (TaKaRa, Otsu, Japan), with an initial step of 94°C for 3 min followed by 20–35 cycles at 94°C for 30 s, 58°C for 30 s and 72°C for 30 s, on the GeneAmp PCR system 9600 (Perkin Elmer-Cetus). Primer sequences for analysing expression patterns and splice-forms of P2XM were E1F (5′-GGCTCCCCAGGGGCTACGACA-3′) and 261R (5′-CCTTGATCTGAGTGACGGAA-3′) for the AL1 region, and 811F (5′-TGCTGGCCTCACTACTCCTT-3′) and 1259R (5′-GGTTGGCAAGTGGGTGTCAG-3′) for the AL2 and AL3 regions together. For analysing the coding region of mdm2 transcripts, PCR was performed by 35 cycles at 94°C for 30 s, 55°C for 30 s and 72°C for 3 min using a sense primer (5′-GATGGTGCTGTAACCACCTC-3′) and an antisense primer (5′-CTACTAGAAGTTGATGGCTGAG-3′) and 665-bp PCR products were resolved by electrophoresis in 2% agarose gels.

### PCR-SSCP screening for p53 mutations

A total of 100 ng of each tumour DNA were amplified by the PCR using Ex Taq and 5 pM of each primer in 35 cycles under the cycling conditions described for the reverse transcription-PCR (RT-PCR) of P2XM. The primer sequences designed to amplify each exon from 5 to 8 and parts of adjacent introns were E5F (5′-CTTGTGCCCTGACTTTCAAC-3′) and E5R (5′-TCTCCAGCCCCAGCTGCTC-3′) for exon 5; E6F (5′-TGATTCCTCACTGATTGCTCT-3′) and E6R (5′-CCAGAGACCCCAGTTGCAAAC-3′) for exon 6; E7F (5′-TCTTGGGCCTGTGTTATCTC-3′) and E7R (5′-TGCAGGGTGGCAAGTGGCTCC-3′) for exon 7; and E8F (5′-GCTTCTCTTTTCCTATCCTGA-3′) and E8R (5′-ACCGCTTCTTGTCCTGCTTGC-3′) for exon 8. We performed non-radioactive single strand conformation polymorphism (SSCP) analysis using a FMBIOII Multiview fluorescent image analyser (TaKaRa, Otsu, Japan). A 2.5-μl aliquot of PCR product from each sample was mixed with SSCP-loading buffer that contained 95% formamide, 10 mM EDTA, 0.25% bromophenol blue and xylenecyanol, boiled for 5 min and placed on ice. Twenty microlitres of each mixture was electrophoresed with 0.5% TBE buffer for 12 h in a 5% polyacrylamide gel containing 10% glycerol, at a continuous 500 volts, in a chromatography chamber maintained at 4°C. After electrophoresis, the gels were stained for 5 min with SYBR GREEN II (FMC Bioproducts, Rockland, ME, USA) diluted 1/100 000 with 0.5 × TBE, and visualized by scanning with the FMBIOII imager. Aberrant bands were extracted from the gels into 500 μl of TE and boiled for 30 min; 1 μl of this solution was used for the second PCR amplification.

### Cloning and sequencing of extracted DNA fragments

The second PCR for each extracted DNA fragment was performed using the same primers and conditions as in the first PCR. Amplified products were electrophoresed in 2% agarose gels; each band was excised and its DNA extracted using the Qiagen (Santa Clarita, CA, USA) DNA extraction kit. The DNA fragments were blunt-ended and cloned into the *Eco*RV site of pBluescript II (Stratagene, La Jolla, CA, USA). Five clones of each fragment were sequenced to avoid errors due to mis-incorporation of *Taq* polymerase or contamination by PCR products amplified from retained or coexistent normal alleles. Sequencing was performed in an ABI model 373 auto-sequencer (Perkin Elmer-Cetus) by the dideoxy chain-termination method, using the AmpliTaq FS cycle-sequencing kit (Applied Biosystems, Foster City, CA, USA).

### RESULTS

### Analysis of P2XM expression

In our previous study of P2XM expression in cell lines derived from soft-tissue or bone tumours we noted, in addition to the full-size transcript, three splicing variants which lacked parts of, or entire, exons (Figure 1). The first alternatively spliced form, AL1, lacked a 78-bp portion of exon 1 which would encode part of the M1 transmembrane domain. The second variant, AL2, deleted exon 10 (66-bp); exons 10 and 11 (144-bp) were both eliminated in the third splicing variant, AL3. As the M2 transmembrane domain would be encoded by exons 10 and 11, the proteins translated from AL2 and AL3 would have no intact M2 moiety. In the present study, we performed RT-PCR using primers to amplify separate regions (N1 and N2) spanning the M1 or M2 transmembrane domains respectively, in normal muscle and in a large panel of primary soft-tissue tumours. In normal skeletal muscle, the 155-bp AL1 transcript is expressed at a level significantly lower than that of the 233-bp normal (N1) transcript (Figure 2). Similarly, AL2 (402-bp) and AL3 (324-bp) transcripts were expressed in much lower quantities than the 468-bp normal transcript of this

© 1999 Cancer Research Campaign

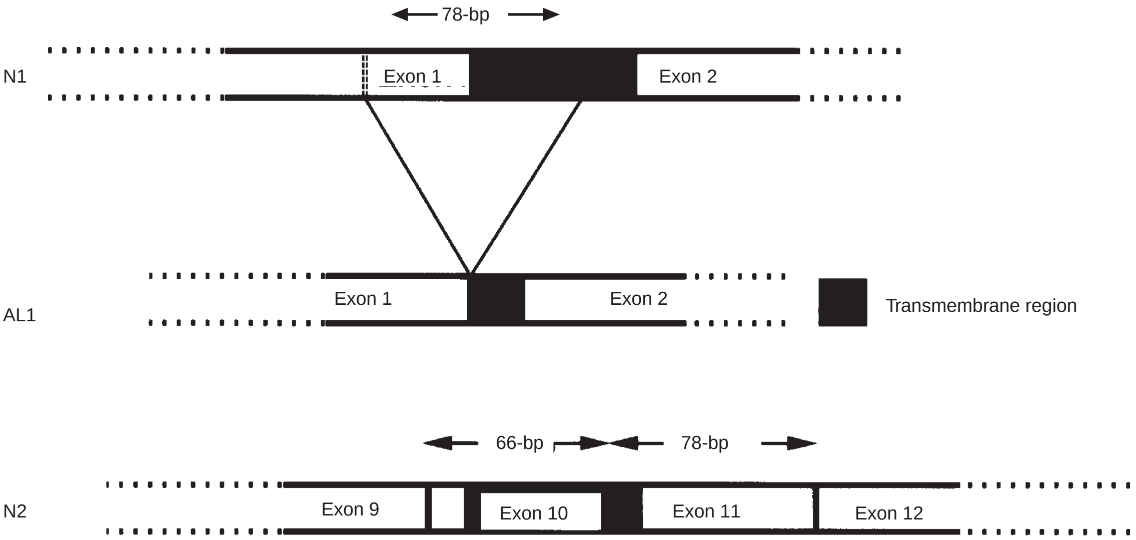

**Figure 1** Schematic presentation of alternatively spliced variants. N1, normal transcript of the 5′ portion of P2XM containing M1. AL1 (alternative splicing 1) lacks part of the transmembrane domain encoded in exon 1. Alternative splice-forms 2 (AL2) and 3 (AL3) respectively lack exon(s) 10 and 10–11 of the normal transcript (N2) that encodes M2

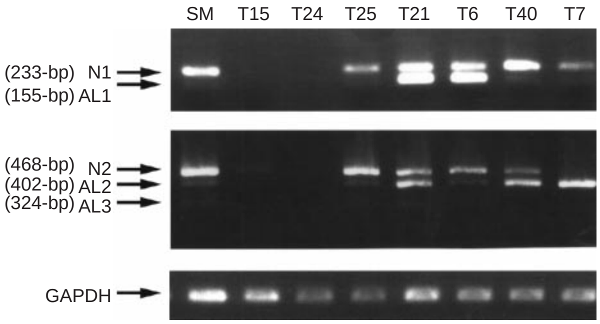

**Figure 2** RT-PCR products of regions N1 and N2 of P2XM, containing the M1 and M2 domains respectively, in several soft-tissue tumours. SM, normal skeletal muscle; T15, leiomyosarcoma; T24, rhabdomyosarcoma; T25 and T21, MFHs; T6, T40 and T7, liposarcomas

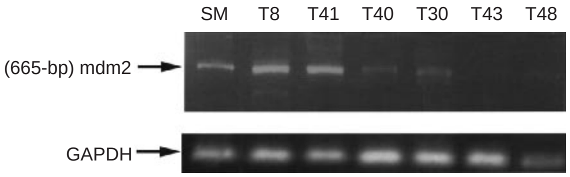

**Figure 3** RT-PCR products corresponding to a part of coding region of mdm2. In a comparision to normal skeletal muscle (SM), increased products were demonstrated in lanes amplified from T8 and T41. SM, normal skeletal muscle; T8, T40, T41 and T43, liposarcomas; T30 and T48, MFHs region (N2; Figure 2). Northern blott analysis with poly-adenosine (poly-A) RNA derived from multiple tissues detected only the full-sized P2XM transcript in skeletal muscle (data not shown); this result supported the view that the alternatively spliced forms are normally expressed at very low abundance. We analysed expression of P2XM in 47 STSs and nine benign tumours, and judged that expression was lost (LOE) when neither the normal transcript nor any splicing variant was detectable on agarose gel electrophoresis after 35 cycles of PCR amplification in the regions corresponding to M1 and M2 (for example, T15 and T24 in Figure 2). When any splicing variant (AL1, 2, or 3) was expressed more abundantly than the normal transcript (e.g. T21, T6, T40, and T7 in Figure 2), we scored such tumours as aberrant transcript-dominant (ATD). As summarized in Table 1, 34 (72%) of the 47 sarcomas we examined revealed aberrant expression patterns of P2XM; 12 cases showed the LOE pattern and 22 had the ATD pattern. Even the majority of benign tumours revealed aberrant expression of P2XM; among the nine such tumors examined, four showed the LOE pattern and three the ATD pattern. We found no correlation between histopathological type and the pattern of P2XM expression. None of the tumours examined produced abundant AL3, but abundant expression of AL1 and AL2 variants was observed in 16 (34%) and 13 (28%) of the 47 sarcomas respectively. Nine tumours (seven malignant and two benign) revealed elevated expression of transcripts that exhibited both AL1 and AL2 types of variation, while 16 others expressed transcripts involving alteration of only one region.

### Screening of p53 mutations

We analysed the same panel of tumours for mutations in the p53 gene, specifically in exonic and flanking intronic sequences covering exons 5–8 where most p53 mutations tend to be clustered. PCR-SSCP analysis detected nine aberrant bands in eight tumours, including five of the MFHs, one liposarcoma, one synovial sarcoma and one lipoma. DNA sequencing of these bands disclosed seven different mis-sense mutations, one non-sense mutation, and a 6-bp deletion (Table 2). The liposarcoma (T7) contained both a mis-sense mutation and a 6-bp deletion. All nine mutations were confirmed by repeated experiments.

Among the eight tumours carrying mutant p53 alleles, all except one MFH (T25) had shown aberrant expression of P2XM; in five of them, expression of splice-form(s) was elevated and in the other two P2XM expression was lost altogether.

© 1999 Cancer Research Campaign

**Table 1** Expression pattern of *P2XM* and *p53/mdm2* status in soft tissue tumours

| Histological type | Number of cases | Loss of expression | Abundant splicing variants AL1 | AL2 | AL1 and/or AL2 | Total no. *P2XM* | *p53* mutation or *mdm2* overexpression |
|---|---|---|---|---|---|---|---|
| Soft tissue sarcoma | | | | | | | |
| MFH | 15 | 3 | 5 | 5 | 7 | 10 | 5 |
| Liposarcoma | 9 | 1 | 3 | 2 | 5 | 6 | 3 |
| Synovial sarcoma | 7 | 4 | 3 | 2 | 3 | 7 | 2 |
| Malignant schwannoma | 6 | 1 | 2 | 2 | 3 | 4 | 0 |
| Leiomyosarcoma | 3 | 1 | 1 | 1 | 2 | 3 | 0 |
| Rhabdomyosarcoma | 2 | 1 | 1 | 1 | 1 | 2 | 0 |
| Alveolar softpart sarcoma | 2 | 0 | 0 | 0 | 0 | 0 | 1 |
| Other and unclassified | 3 | 1 | 1 | 0 | 1 | 2 | 1 |
| Total | 47 | 12 | 16 | 13 | 22 | 34 | 12 |
| Benign tumour | | | | | | | |
| Lipoma | 3 | 1 | 2 | 2 | 2 | 3 | 1 |
| Desmoid tumour | 2 | 1 | 0 | 1 | 1 | 2 | 0 |
| Schwannoma | 2 | 1 | 0 | 0 | 0 | 1 | 0 |
| Other | 2 | 1 | 0 | 0 | 0 | 1 | 0 |
| Total | 9 | 4 | 2 | 3 | 3 | 7 | 1 |
| Total | 56 | 16 | 18 | 16 | 25 | 41 | 13 |

**Table 2** Mutations of *p53* and *mdm2* status in soft tissue sarcomas and benign soft tissue tumours

| Case | Histology | Mutations of *p53* Exon | Codon | Mutation | Resulting change | *mdm2* overexpression | Expression pattern of *P2XM* |
|---|---|---|---|---|---|---|---|
| T7 | Liposarcoma | 7 | 239–241 | 6bp deletion | Asn-Ser-Ser-> Thr | – | Abundance of AL2 |
| | | 7 | 253 | *A*CC->*G*CC | Thr->Ala | | |
| T8 | Liposarcoma | | | – | | + | Abundance of AL2 |
| T41 | Liposarcoma | | | – | | + | Abundance of AL1 |
| T20 | Lipoma | 5 | 169 | *A*TG->*G*TG | Met->Val | – | Abundance of AL1 and AL2 |
| T12 | Synovial sarcoma | 6 | 194 | *C*TT->*T*TT | Leu->Phe | – | Abundance of AL1 and AL2 |
| T13 | Synovial sarcoma | | | – | | + | Loss of expression |
| T22 | MFH | 5 | 175 | C*G*C->C*A*C | Arg->His | – | Loss of expression |
| T25 | MFH | 7 | 241 | *T*CC->*A*CC | Ser->Thr | – | Normal |
| T32 | MFH | 6 | 213 | *C*GA->*T*GA | Stop at codon 213 | – | Loss of expression |
| T55 | MFH | 5 | 126 | T*A*C->T*T*C | Tyr->Phe | – | Abundance of AL1 |
| T56 | MFH | 7 | 248 | C*G*G->C*A*G | Arg->Gln | – | Abundance of AL1 and AL2 |
| T35 | ASPS | | | – | | + | Normal |
| T14 | Unclassified | | | – | | + | Normal |

### Analysis of mdm2 overexpression

In the present study, we also performed semiquantitative RT-PCR for analysing overexpression of mdm2 transcript. We judged that mdm2 was overexpressed when the density of the band corresponding to a part of mdm2 was increased more than five times in a comparison to that of normal skeletal muscle by agarose gel electrophoresis after PCR amplification (Figure 3). Overexpression of mdm2 was observed in five sarcomas, including two liposarcomas, one each of synovial sarcoma, ASPS and unclassified sarcoma which carried wild-type p53. Three out of five tumours with mdm2 overexpression showed abnormal P2XM expression pattern; two ATD and one LOE patterns. Therefore, ten out of 13 soft-tissue tumours with either p53 mutation or mdm2 overexpression demonstrated abnormal expression of P2XM.

### DISCUSSION

In the study reported here, we found that 34 (72%) of the 47 soft-tissue sarcomas we examined exhibited altered expression of the P2XM gene; in 12 cases expression was lost and in 22 cases the majority of the putative gene products were considered to have lost one or both of the transmembrane domains. We found no alterations in either of two alveolar softpart sarcomas, but were unable to demonstrate any significant correlation between P2XM alterations and histological types. In three-fourths of the tumours with aberrant splicing, transcripts containing variants of either the AL1 or AL2 type were expressed predominantly; the rest produced transcripts that included both variations. However, none of the tumours was found to have elevated expression of the AL3 type of transcript previously demonstrated in the cell lines. Although the mechanism involved in alternative splicing of the P2XM gene is not clear, it is certain that the AL1 and/or AL2 transcripts were selectively up-regulated at the transcriptional level in the tumour cells studied here. It is notable that aberrant patterns were also seen in benign tumours such as lipoma, desmoid tumour and schwannoma, and at a similar frequency. These results suggested that the loss of function of P2XM is related to tumorigenesis in various types of soft-tissue tumours, both malignant and benign.

The P2XM gene, a member of the P2X family of receptors that open cationic channels by binding ATP, contains two transmembrane domains and an H5 region characteristic of voltage-gated

 © 1999 Cancer Research Campaign

K+ channels. Furthermore, it bears nucleotide-sequence similarity to RP-2, a gene that is induced when thymocytes are undergoing apoptosis (Owens et al, 1991). These observations may indicate that the function of P2XM is associated with programmed cell death, mediated by ATP or synaptic transmission. Considering that p53 is a key player in the apoptosis pathway, p53-inducible P2XM is likely to be involved in this process.

SSCP analysis and subsequent DNA sequencing disclosed p53 mutations in seven sarcomas and one benign lipoma. The frequency of p53 mutations in our panel of tumours is close to the average frequency generally reported in soft-tissue tumours (10–33%) (Stratton et al, 1990; Toguchida et al, 1992; Taubert et al, 1995, 1996; Würl et al, 1996; Schneider-Stock et al, 1997).

The pattern of P2XM expression was changed in all of the tumours carrying p53 mutations except for one MFH (T25) with a mis-sense mutation at codon 241. In the rest of the tumours with wild-type p53, three sarcomas with mdm2 overexpression (T8, T41 and T13) showed altered pattern of P2XM expression. Hence, in a majority of tumours (31 out of 43) that had wild-type p53 without mdm2 overexpression, P2XM expression was altered, genetic factor(s) other than p53 or mdm2 may contribute for aberrant P2XM expression. Our study suggests that an insufficiency of normal P2XM, as a consequence of being down-regulated due to p53 mutation, mdm2 overexpression or as the result of deregulation of the splicing machinery, could contribute to development and/or progression of the majority of soft-tissue tumours.

### ACKNOWLEDGEMENTS

We thank Mrs Nishijima for her technical assistance. This work was supported by a special grant for Strategic Advanced Research on Cancer from the Ministry of Education, Culture, Sports, and Science of Japan; and a Research for the Future Program Grant (96L00102) of the Japan Society for the Promotion of Science.

### REFERENCES

Andreassen A, Oyjord T, Hovig E, Holm R, Florenes VA, Nesland JM, Myklebost O and Hoie J (1993) p53 abnormalities in different sub-types of human sarcomas. *Cancer Res* **53**: 468–471

Buckbinder L, Talbott R, Velasco MS, Takenaka I, Faha B, Seizinger BR and Kley N (1995) Induction of the growth inhibitor IGF-binding protein 3 by p53. *Nature* **377**: 646–649

Castresana JS, Barrios C, Gómez L and Kreicbergs A (1994) No association between c-myc amplification and tp53 mutation in sarcoma tumorigenesis. *Cancer Genet Cytogenet* **76**: 47–49

Castresana JS, Rubio M-P, Gómez L, Kreicbergs A, Zetterberg A and Barrios C (1995) Detection of tp53 gene mutations in human sarcomas. *Eur J Cancer* **31A**: 735–738

Cordon-Cardo C, Latres E, Drobnjak M, Oliva M, Pollack D, Woodruff JM, Marechal V, Chen J, Brennan MF and Levine AJ (1994) Molecular abnormalities of *mdm2* and *p53* genes in adult soft tissue sarcomas. *Cancer Res* **54**: 794–799

El-Deiry WS, Tokino T, Velculescu VE, Levy DB, Parsons R, Trent JM, Lin D, Mercer WE, Kinzler KW and Vogelstein B (1993) WAF1, a potential mediator of p53 tumor suppression. *Cell* **75**: 817–825

Enjoji M and Hashimoto H (1984) Diagnosis of soft-tissue sarcomas. *Pathol Res Pract* **178**: 215–226

Enzinger FM, Lattes R and Torloni R (1969) Histologic typing of soft-tissue tumors. International classification of tumors 3: WHO, Geneva

Felix CA, Kappel CC, Mitsudomi T, Nau MM, Tsokos M, Crouch GD, Nisen PD, Winick NJ and Helman IJ (1992) Frequency and diversity of p53 mutations in childhood rhabdomyosarcoma. *Cancer Res* **52**: 2243–2247

Furuhata T, Tokino T, Urano T and Nakamura Y (1996) Isolation of a novel GPI-anchored gene specifically regulated by p53; correlation between its expression and anticancer drug sensitivity. *Oncogene* **13**: 1965–1970

Haupt Y, Maya R, Kazaz A and Oren M (1997) Mdm2 promotes the rapid degradation of p53. *Nature* **387**: 296–9

Kastan MB, Zhan Q, El-Deiry WS, Carrier F, Jacks T, Walsh WV, Plunkett BS, Vogelstein B and Fornace AJ (1992) A mammalian cell cycle checkpoint pathway utilizing p53 and GADD45 is defective in ataxia-telangiectasia. *Cell* **71**: 587–597

Kubbutat MH, Jones SN and Vousden KH (1997) Regulation of p53 stability by Mdm2. *Nature* **387**: 299–303

Latres E, Drobnjak M, Pollack D, Oliva MR, Ramos M, Karpeh M, Woodruff JM and Cordon-Cardo C (1994) Chromosome-17 abnormalities and tp53 mutations in adult soft-tissue sarcoma. *Am J Pathol* **145**: 345–355

Leach FS, Tokino T, Burrell M, Oliner JD, Smith S, Hill DE, Sidransky D, Kinzler KW and Vogelstein B (1993) p53 mutations and mdm2 amplification in human soft-tissue sarcomas. *Cancer Res* **53**: 2231–2234

Masuda H, Miller C, Koeffler H, Battifora H and Cline M (1987) Rearrangement of the p53 gene in human osteogenic sarcomas. *Proc Natl Acad Sci USA* **84**: 7716–7719

Miyashita T and Reed JC (1995) Tumor suppressor p53 is a direct transcriptional activator of the human bax gene. *Cell* **80**: 293–299

Morris GE, Bischoff JR and Mathews MB (1996) Transcriptional activation of the human proliferating cell nuclear antigen promoter by p53. *Proc Natl Acad Sci USA* **93**: 895–899

Okamoto K and Beach D (1994) Cyclin G is a transcriptional target of p53 tumor suppressor protein. *EMBO J* **13**: 4816–4822

Oliner JD, Kinzler KW, Meltzler PS, George DL and Vogelstein B (1992) Amplification of a gene encoding a p53-associated protein in human sarcomas. *Nature* **358**: 80–83

Owens GP, Hahn WE and Cohen JJ (1991) Identification of mRNAs associated with programmed cell death in immature thymocytes. *Mol Cel Biol* **11**: 4177–4188

Schneider-Stock R, Radig K, Oda Y, Mellin W, Rys J, Niezabitowski A and Roessner A (1997) p53 gene mutations in soft-tissue sarcomas – correlations with p53 immunohistochemistry and DNA ploidy. *J Cancer Res Clin Oncol* **123**: 211–218

Stratton MR, Moss S, Warren W, Patterson H, Clark J, Fisher C, Fletcher C, Ball A, Thomas M, Gusterson B and Cooper C (1990) Mutation of the p53 gene in human soft-tissue sarcomas: association with abnormalities of the Rb1 gene. *Oncogene* **5**: 1297–1301

Taubert H, Würl P, Meye A, Berger D, Thamm B, Neumann K, Hinze R, Schmidt H and Rath FW (1995) Molecular and immunohistochemical p53 status in liposarcoma and malignant fibrous histiocytoma. *Cancer* **76**: 1187–1195

Taubert H, Meye A and Würl P (1996) Prognosis is correlated with p53 mutation type for soft tissue sarcoma patients. *Cancer Res* **56**: 4134–4136

Toguchida J, Yamaguchi T, Ritchie B, Beauchamp RL, Dayton SH and Herrera GE (1992) Mutation spectrum of the p53 gene in bone and soft-tissue sarcomas. *Cancer Res* **52**: 6194–6199

Urano T, Nishimori H, Han H, Furuhata T, Kimura Y, Nakamura Y and Tokino T (1997) Cloning of P2XM, a novel human P2X receptor gene regulated by p53. *Cancer Res* **57**: 3281–3287

Vogelstein B and Kinzler KW (1992) p53 function and dysfunction. *Cell* **70**: 523–526

Wu X, Bayle JH, Olson D and Levine AJ (1993) The p53-mdm-2 autoregulatory feedback loop. *Genes Devel* **7**: 1126–1132

Würl P, Taubert H, Bache M, Kroll J, Meye A, Berger D, Siermann A, Holzhausen H-J, Hinze R, Schmidt H and Rath F-W (1996) Frequent occurrence of p53 mutations in rhabdomyosarcoma and leiomyosarcoma, but not in fibrosarcoma and malignant neural tumors. *Int J Cancer* **69**: 317–323

© 1999 Cancer Research Campaign
